# Machine learning-based prediction of heat transfer performance in annular fins with functionally graded materials

**DOI:** 10.1038/s41598-024-58595-6

**Published:** 2024-04-16

**Authors:** Muhammad Sulaiman, Osamah Ibrahim Khalaf, Naveed Ahmad Khan, Fahad Sameer Alshammari, Sameer Algburi, Habib Hamam

**Affiliations:** 1 Department of Mathematics, Abdul Wali Khan University, 23200 Mardan, Pakistan; 2 Department of Solar, Al-Nahrain Research Center for Renewable Energy, Al-Nahrain University, Jadriya, Baghdad, Iraq; 3 School of Information Technology and Systems, University of Canberra, Canberra, ACT Australia; 4 Academy of Mathematics and Systems Science, Chinese Academy of Sciences, Beijing, China; 5 Department of Mathematics, College of Science and Humanities in Alkharj, Prince Sattam bin Abdulaziz University, Al-Kharj, 11942 Saudi Arabia; 6 Department of Computer Engineering Technologies, Al-Kitab University, Kirkuk, 36015 Iraq; 7 Faculty of Engineering, Uni de Moncton, Moncton, NB E1A3E9 Canada; 8 Hodmas University College, Taleh Area, Mogadishu, Somalia; 9 Bridges for Academic Excellence, Tunis, Centre-Ville, Tunisia; 10 School of Electrical Engineering, University of Johannesburg, Johannesburg, South Africa

**Keywords:** Funtionally graded fin, Heat transfer, Temperature distribution, Machine learning, Thermal analysis, Supervised neural networks, Engineering, Mathematics and computing

## Abstract

This paper presents a study investigating the performance of functionally graded material (FGM) annular fins in heat transfer applications. An annular fin is a circular or annular structure used to improve heat transfer in various systems such as heat exchangers, electronic cooling systems, and power generation equipment. The main objective of this study is to analyze the efficiency of the ring fin in terms of heat transfer and temperature distribution. The fin surfaces are exposed to convection and radiation to dissipate heat. A supervised machine learning method was used to study the heat transfer characteristics and temperature distribution in the annular fin. In particular, a feedback architecture with the BFGS Quasi-Newton training algorithm (trainbfg) was used to analyze the solutions of the mathematical model governing the problem. This approach allows an in-depth study of the performance of fins, taking into account various physical parameters that affect its performance. To ensure the accuracy of the obtained solutions, a comparative analysis was performed using guided machine learning. The results were compared with those obtained by conventional methods such as the homotopy perturbation method, the finite difference method, and the Runge–Kutta method. In addition, a thorough statistical analysis was performed to confirm the reliability of the solutions. The results of this study provide valuable information on the behavior and performance of annular fins made from functionally graded materials. These findings contribute to the design and optimization of heat transfer systems, enabling better heat management and efficient use of available space.

## Introduction

Fins play a crucial role in thermal management by acting as extended surfaces that facilitate the transfer of excess heat from the primary surface, such as equipment, to the surrounding environment. This transfer mechanism is necessary to maintain the desired operating temperature of the primary surface^1^. The effectiveness of the rows lies in their ability to increase the available surface for heat exchange with the adjacent refrigerant, which increases the rate of heat transfer. Therefore, fins are widely used as cooling components in various devices and systems, including internal combustion engines, compressors, heat exchangers, transformers, nuclear rods, space batteries, and electronic equipment^2^. These applications rely on fins to efficiently remove heat and prevent negative effects associated with excessive temperatures. Of particular interest among the different types of fins are annular fins, which have received considerable attention in current research due to their unique compact design. Annular fins offer distinct advantages over similarly sized straight fins, primarily due to their ability to provide a greater surface area for the coolant^3^. This feature improves heat transfer performance. In addition, the compact structure of ring fins makes them very suitable for use where space is limited, for example in tube heat exchangers. The ease of fabrication of standard thick ring fins further enhances their appeal as a practical choice for such heat transfer systems^4^.

Recently, there is a growing demand for fins with features such as lighter weight, faster heat transfer rates, and better structural stability. This has increased the attractiveness of modern materials over traditional materials traditionally used in rib applications^5^. However, it is worth noting that most research on the fin has focused on conventional materials, mainly due to the mathematical complexity involved in the analysis and the limitations of the manufacturing processes associated with modern materials. Despite these challenges, researchers have begun to explore the potential of functionally graded materials (FGM) in array design^6^. FGMs are materials whose composition and properties change gradually, allowing tailored thermal properties. Although previous research on FGM fins has mostly focused on conduction-convection fins, where the conduction parameter was assumed to be a function of fin length, it is important to consider other important thermal parameters that cannot be neglected^7^. For example, Campo and Wolko made a study of rectangular fins with temperature-dependent power law variations of thermal conductivity and emissivity^8^. They noted that it was important to include these differences in the thermal properties to accurately assess the heat transfer efficiency of the fin. Similarly, Lesnic and Heggs used the decomposition method to study the temperature distribution in a straight fin with a temperature-dependent heat transfer coefficient^9^ power variation. By considering the variable heat transfer coefficient, they aim to obtain a more comprehensive understanding of the temperature distribution of the fin. These studies highlight the need to investigate and include different thermal parameters in the analysis of FGM fins. By considering temperature-dependent properties such as thermal conductivity, emissivity, and heat transfer coefficients, a more accurate representation of heat transfer behavior can be obtained. This is critical in efforts to design fins that meet increased performance requirements, including lighter weight, faster heat transfer rates, and improved structural stability. Although the mathematical complexity and manufacturing limitations associated with today’s materials still present challenges, research on FGM fins is a promising tool for advancing fin technology. By developing a deeper understanding of the thermal properties and behavior of female genital fins, researchers and engineers can pave the way for the development of innovative heat transfer solutions that optimize efficiency and meet the demands of modern applications^10^.

Many nonlinear equations related to scientific and engineering problems do not have analytical solutions, so numerical methods are required to solve them. Various techniques have been developed to address this challenge, including semi-analytical methods such as Adomian decomposition, differential transform, and hybrid differential transform methods (DTMs). These methods have proven to be elegant approaches to solving nonlinear equations. In addition to these semianalytical methods, classical numerical techniques such as finite difference, finite volume, and finite element methods are commonly used to solve such equations. Chiu and Chen^11^ conducted a study using the Adomian double decomposition method to analyze the temperature distribution and estimate the stress field in a circular fin with different thermal conductivities. In a separate study, Malekzadeh^12^ used the differential quadrature element method to study a convective rib with different thermal conductivity, highlighting its computational efficiency, high accuracy, and convergence speed. Recently, Peng and Chen^13^ used a hybrid numerical approach combining differential transformation and finite difference methods to solve the heat transfer equation of an annular fin with temperature-dependent thermal conductivity. Their model considered the simultaneous removal of heat by convection and radiation from the surface of the fin to the surrounding medium. The study investigated the effect of various thermal parameters such as heat transfer coefficient, absorptivity, emissivity and thermal conductivity on the temperature distribution. Sharma and Hanumagowda^14^ conducted a study on radial annular fins, considering the effects of a ternary nanofluid, magnetic field, permeable medium, and thermal radiation. They employed the differential transformation method to obtain an analytical solution and demonstrated how nondimensional characteristics influence the thermal gradient of the fin. Aksoy used the homotopy analysis method (HAM)^15^ to investigate the thermal efficiency of an annular fin considering variable heat transfer coefficient and thermal conductivity. It is important to note that these previous studies did not consider heat generation. However, in certain cases, internal heat generation cannot be ignored in heat transfer processes, especially at high temperatures. Hossain Nemati and Sina Samivand^16,17^ proposed a simple correlation to evaluate the efficiency of annular elliptical fins circumscribing circular tubes. Their study revealed that constant temperature lines deviate from circular patterns in lower aspect ratio fins, affecting the accuracy of common methods like equivalent fin area or sector methods. Aziz and Bouaziz^18^ tried to solve a nonlinear heat transfer equation with different thermal conductivity and heat transfer in the longitudinal direction using the least square method. They found that internal heat production affected temperature distribution and efficiency. Similarly, Georgiou and Razelos^19^ investigated the effect of internal heat generation on the performance of a convective annular fin with a trapezoidal profile and showed a degradation of the thermal convective capacity of the fin due to internal heat generation. Some researchers have also considered a temperature-dependent heat transfer parameter, which provides a more realistic representation of the phenomenon.

These methods have been effectively applied to study the behaviors and characteristics of the problem but besides there advantages there are certain limitations. Adomian’s Double Decomposition Method, although effective in some cases, may face challenges when dealing with highly nonlinear equations or complex boundary conditions. Convergence of the method can be difficult to achieve in such scenarios, requiring careful analysis and potentially limiting its applicability^20^. The Differential Quadrature Element Method offers computational efficiency and high accuracy, making it an attractive choice. However, this method may encounter difficulties when applied to problems with irregular or complex geometries, as discretization and element arrangement become more challenging. Moreover, large-scale problems may require substantial computational resources, which can be a limitation in practical implementations^21^. Hybrid numerical methods combining different techniques, such as the combination of differential transformation and finite difference methods, can provide advantages in certain cases. However, these hybrid methods introduce additional complexities in terms of implementation and computational effort. The accuracy and stability of the combined approach need to be carefully assessed to ensure reliable results^22^. The least squares method, employed to address nonlinear heat transfer equations with heat generation, brings its own set of considerations. The choice of appropriate weighting functions and the formulation of the least squares problem play a crucial role in achieving accurate and reliable solutions. Finally, the homotopy analysis method (HAM) used in several studies can show slower convergence rates for highly nonlinear problems. Researchers must carefully determine the appropriate auxiliary parameters and access criteria to ensure the effectiveness and efficiency of the method^23^. Given these limitations, researchers must thoroughly evaluate the applicability of each method based on the problem at hand, including the nature of the problem, boundary conditions, and available computational resources.

The development of machine learning algorithms was crucial to address the shortcomings and difficulties of traditional methods. Machine learning solves problems using a data-driven approach, where algorithms learn from training data and make predictions based on patterns they see. By studying large data sets, these algorithms are able to adapt and improve their performance. Using machine learning, researchers can solve complex questions in many different fields, including physics, biology and engineering^24^. Machine learning algorithms are very successful in solving complex geometries, non-linear equations and large-scale problems. They can independently extract the most important features and identify links in the data, enabling insights and predictions that were difficult to make using traditional approaches. In addition, machine learning approaches can be optimized and computationally efficient, allowing researchers to make accurate predictions and successfully solve complex problems in a timely and reliable manner. Some possible applications of machine learning algorithms include the transition of disturbances due to changes in braking and acceleration in traffic flows^25^, heat transfer in a micropolar fluid with isothermal and isoflux boundary conditions^26^, stiffness solutions for polytropic. gas spheres and electric circuit models^27^, linearization of circular sector oscillator inelastic vibration analysis^28^ and inclined longitudinal porous trapezoidal and rectangular trapezoidal fin heat transfer analysis.

## Problem discription

**Figure 1:**
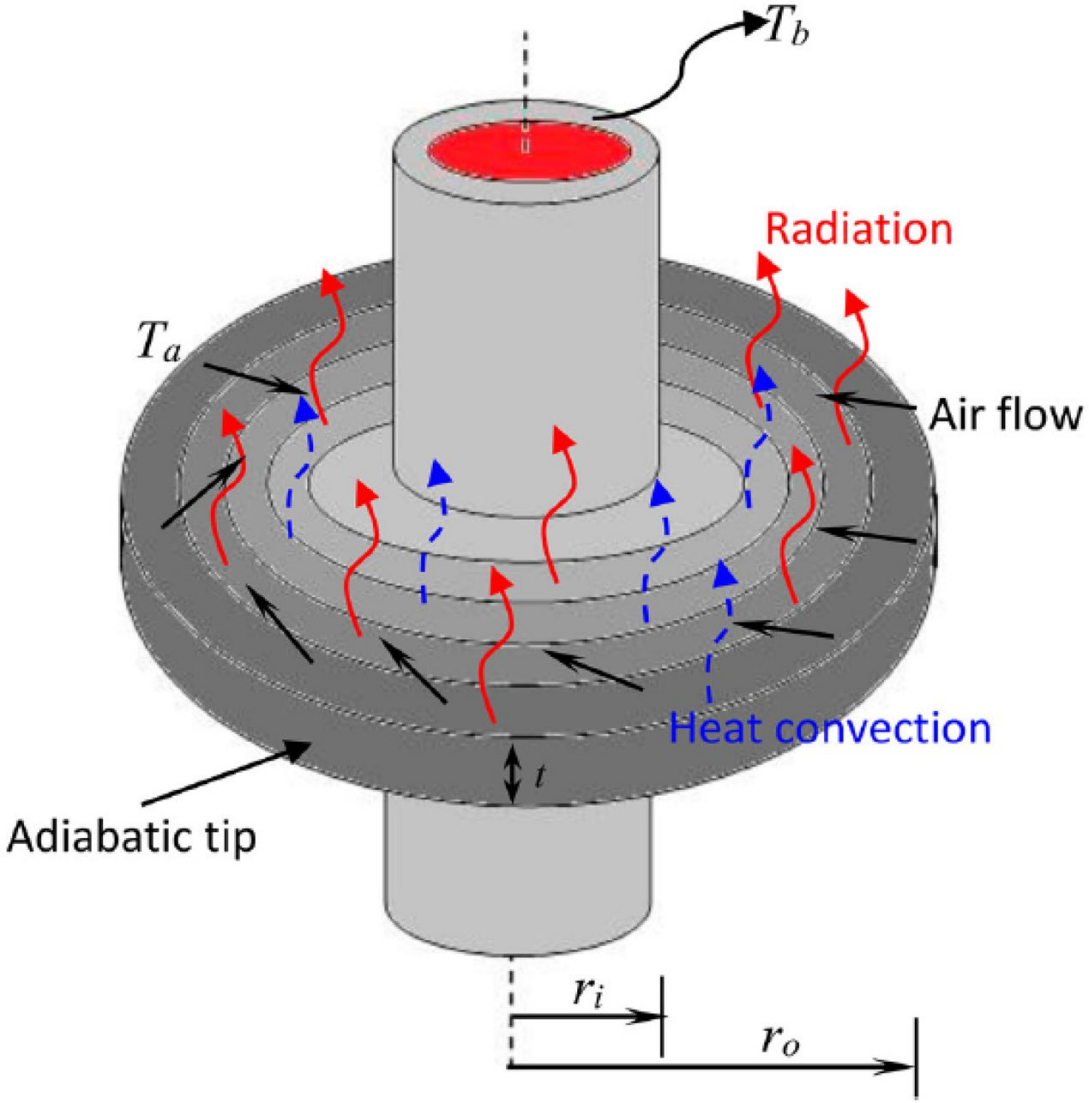
Geometric configuration and visualization of a heat transfer-optimized annular fin incorporating functionally graded material.

The objective of this study is to analyze the heat transfer capabilities of an annular fin made of functionally graded material (FGM). Figure 1 shows the fin, which has three dimensions: an outer radius $(r_o)$, an inner radius $(r_i)$, and a thickness (*t*). The main objective is to maximize heat dissipation from the heated body while maximizing heat transmission from the surface of the fin to the surroundings. The tip of the fin is thought to be well-insulated whereas the rest of it is exposed to a base temperature $(T_b)$. Convection and radiation work together to transfer heat. $T_a$ stands for the surrounding temperature for convection, and $T_s$ for the surrounding temperature for radiation. The emissivity $(\epsilon )$ of the fin material displays a nonlinear variation with *r*, but the thermal conductivity (*k*) of the fin material exhibits a linear variation with the spatial coordinate (*r*). Additionally, only temperature (*T*) affects the internal heat production (*q*) and the convective heat transfer coefficient (*h*).

The Fourier’s law of heat conduction’s guiding principles can be used to analyse the current problem. The energy balance equation and its accompanying boundary conditions can be written as Eqs. (1) and (2) when the steady-state condition is taken into account. Equation (1) is based on the assumption of gray and diffuse material. The fin is supposed to have good insulation throughout, little heat loss at the base, and a well-insulated tip.

1 $$\begin{aligned}{} & {} t \frac{\text{d}}{\text{d} r}\left[ \{k(r)\} r \cdot \frac{\text{d} T}{\,\text{d} r}\right] -2 \varepsilon (r) \sigma r\left( T^4-T_s^4\right) -2 \left( T-T_a\right) h(T) r+q(T)tr=0, \end{aligned}$$

2 $$\begin{aligned}{} & {} T=T_b \quad \text {at}\quad r=r_i \quad \text {and}\quad \frac{\text{d} T}{\,\text{d} r}=0 \quad \text {at}\quad r=r_{\text{o}}. \end{aligned}$$

The mathematical relationships based on the assumptions used for the modification of the corresponding thermal parameters are stated below:

3 $$\begin{aligned} k&=k_{\text{o}}\left\{ 1+\gamma \left( \frac{r-r_i}{r_o-r_i}\right) \right\} , \quad h=h_{\text{b}}\left( \frac{T-T_{\text{a}}}{T_{\text{b}} -T_{\text{a}}}\right) ^{m_1}, \varepsilon =\varepsilon _{\text{s}} \left( \frac{r}{r_i}\right) ^{m_2} \text { and } \nonumber \\ q&=q_{\text{o}}\left\{ 1+e\left( T-T_{\text{a}}\right) \right\} \end{aligned}$$

Here, the thermal conductivity at the base of the fin is represented by $k_{\text{o}}$, while $h_{\text{b}}$ corresponds to the convective heat transfer coefficient, which measures the heat exchange between the temperature difference $T_{\text{b}}-T_{\text{a}}$. The surface emissivity at the base of the fin is denoted by $\epsilon _{\text{s}}$, and $q_{\text{o}}$ represents the internal heat generation occurring at the ambient temperature $T_{\text{a}}$. Additionally, the parameter $\gamma$ represents the degree of variation, that characterizes the variation in thermal conductivity of functionally graded materials (FGMs). This parameter plays a significant role in influencing the thermal behavior of the fin. Similarly, *e* captures the variation in internal heat generation. The power law indices, $m_1$ and $m_2$, are associated with the variations in the convective and radiative heat transfer coefficients, respectively. The value of $m_1$ provides valuable information about the specific heat transfer mode. Furthermore, the radius of the fin at any given point is denoted as *r*, and $\sigma$ represents the Stefan–Boltzmann constant, a fundamental constant in thermodynamics. The use of dimensionless parameters is employed to promote simplicity and assure higher applicability.

4 $$\begin{aligned} \theta&=\frac{T}{T_{\text{b}}}, \quad \theta _{\text{a}}=\frac{T_{\text{a}}}{T_{\text{b}}}, \quad \theta _{\text{s}}=\frac{T_{\text{s}}}{T_{\text{b}}}, \quad \xi =\frac{r-r_i}{r_i}, \quad R=\frac{r_{\text{o}}}{r_i}, \quad N_{\text{c}}^2=\frac{2 h_{\text{b}} r_i^2}{k_{\text{o}} t}, \quad \delta =\frac{t}{r_i}, \nonumber \\ N_{\text{r}}&=\frac{2 \sigma \varepsilon _{\text{s}} r_i^2 T_{\text{b}}^3}{k_{\text{o}} t}, \quad G=\frac{q_{\text{o}} r_i^2}{k_{\text{o}} T_{\text{b}}}, \quad \text { and } \quad E_{\text{G}}=e T_{\text{b}}, \end{aligned}$$

where $\xi$ is a dimensionless radius, and $\theta$ is the temperature field that changes with it. The non-dimensional convective environment temperature and the non-dimensional surrounding temperature for radiation are denoted by the parameters $\theta _{\text{a}}$ and $\theta _{\text{S}}$, respectively. The annular ratio *R*, alternate numbers for convection and radiation conduction ($N_{\text{c}}$ and $N_{\text{r}}$), thickness ratio ($\delta$), heat generation (*G*) number, and non-dimensional parameter $E_{\text{G}}$ that characterises variation in heat generation are also present. We may describe the energy balance equation and the associated boundary conditions in the following dimensionless form by inserting these non-dimensional terms.

5 $$\begin{aligned}&\frac{\text{d}^2 \theta }{\text{d} \xi ^2}+\frac{\gamma \xi }{(R-1)} \frac{d^2 \theta }{\text{d} \xi ^2}+\frac{1}{(1+\xi )} \frac{\text{d} \theta }{\text{d} \xi }+\frac{\gamma }{(R-1)} \frac{\text{d} \theta }{\text{d} \xi }+\frac{\xi }{(1+\xi )}\frac{\gamma }{(R-1)} \frac{\text{d} \theta }{\text{d} \xi } \nonumber \\&\qquad -N_{\text{c}}^2 \frac{\left( \theta -\theta _{\text{a}} \right) ^{\left( m_1+1\right) }}{\left( 1-\theta _{\text{a}}\right) ^{m_1}} \nonumber \\&\qquad -N_{\text{r}}(1+\xi )^{m_2}\left( \theta ^4 -\theta _{\text{s}}^4\right) +G\left\{ 1+E_{\text{G}} \left( \theta -\theta _{\text{a}}\right) \right\} =0,\nonumber \\&\quad \theta =1 \text { at } \xi =0 \text { and } \frac{\text{d} \theta }{\text{d} \xi }=0 \text { at } \xi =R-1. \end{aligned}$$

## Proposed methodological framework

In this section, we present the designed methodology used to address the solutions for the heat transfer performance in annular fins, utilizing the feedforward backpropagation neural network (FFBPNN). The feedforward backpropagation neural network is a widely employed neural network architecture that allows for supervised learning and has proven to be effective in various applications, including pattern recognition and function approximation. The FFBPNN comprising interconnected nodes or “neurons” organized into multiple layers. This network operates in a feed-forward manner, where data flows unidirectionally from the input layer, through the hidden layers, and eventually reaches the output layer. Unlike networks that incorporate loops or feedback connections, the FFBPNN is solely focused on transmitting information forward within the network. Let *N* represent the entire number of layers in the network, including the input and output layers. Each layer *L* (where $1 \le L \le N$) is made up of $n_L$ neurons. The activation of the neurons in the input layer $(L=1)$ is simply the input data. For each subsequent layer $L(2 \le L \le N)$, the weighted sum of inputs $z_i^{(L)}$ to the *i* th neuron in layer *L* is computed as the sum of the products between the weights $w_{i j}^{(L)}$ and the activations $a_j^{(L-1)}$ of the neurons in the previous layer, added with the bias $b_i^{(L)}$. This can be expressed as:

6 $$\begin{aligned} z_i^{(L)}=\sum _{j=1}^{n_{L-1}} w_{i j}^{(L)} \cdot a_j^{(L-1)}+b_i^{(L)}. \end{aligned}$$

The activation $a_i^{(L)}$ of the *i* th neuron in layer *L* is obtained by applying an activation function *g* to the weighted sum $z_i^{(L)}$ :

7 $$\begin{aligned} a_i^{(L)}=g\left( z_i^{(L)}\right) , \end{aligned}$$

**Figure 2:**
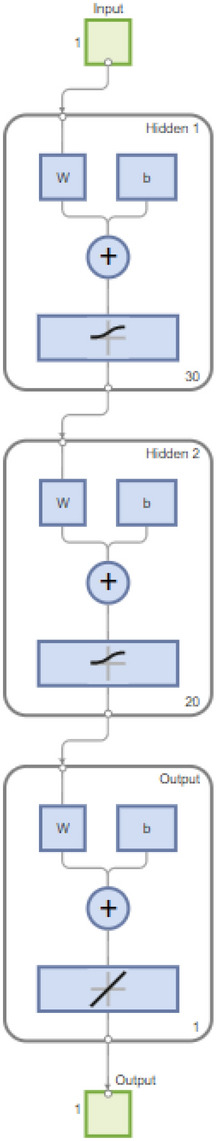
Structure of the FFBPNN model.

In this work, the complex interactions between the input and output variables were examined using the Logsigmoid activation function. The capacity to bring critical non-linearity into the network, allowing for the approximation of complicated nonlinear patterns, and greatly boosting the network’s learning capacities led to the deliberate selection of the logsigmoid activation function as the activation function of preference. The neural network skillfully transferred the weighted sum of inputs to a smooth sigmoidal curve by applying the logsigmoid activation function to each individual neuron in the network, yielding output values within the normalized range. The use of logsigmoid was especially beneficial for binary classification tasks since it allowed decision-making based on programmable threshold values by converting the network’s output into a probabilistic interpretation. Notably, the logsigmoid activation function was crucial in helping the neural network effectively represent and understand the complex relationships present in the dataset, which helped the network achieve remarkable predictive performance and accurate estimations. The structure of the FFBPNN model used in this work is shown in Fig.  2.

The next step is to define a loss function to quantify the difference between the predicted and target output values. The mean squared error (MSE) loss function is used in this scenario. Let $\theta$ represent the desired output and $\hat{\theta }$ represent the expected output for a training sample. The mean squared error is determined as follows:

8 $$\begin{aligned} J=\frac{1}{2 m} \sum _{i=1}^m\left( \hat{\theta }_i-\theta _i\right) ^2, \end{aligned}$$

where *m* is the number of training samples.

### Data sets

The data collection method was carried out to obtain a complete understanding of the heat transfer behavior of the annular fin. This section describes an approach to create a dataset that captures the fin temperature distribution in different scenarios based on the variability of physical parameters such as thermogeometric parameter, convective heat transfer coefficient, conduction-radiation parameter, variable emissivity parameter, and heat production, the heat transfer coefficient, the convective absorption temperature, and the radiative absorption temperature. A nonlinear ordinary differential equation (ODE) was used to simulate the ring fin heat transfer. Equation (5) describes the complex dynamics of the system. The Runge–Kutta method was used to solve the problem. This method of numerical integration is useful for solving ODEs and provides accurate results by repeatedly evaluating the solution over a set of discrete points. The database used in this study contained a total of 2001 individual data points. To evaluate the performance of the machine learning model and determine its generalizability, the dataset was segmented into three distinct subsets: validation, testing, and training. The model is trained by iteratively changing its parameters using training data, which is 70 percent of the total subset. This allows the model to better understand the underlying patterns in the data. The test data, which is 15% of the total data, is used to evaluate the model’s performance on examples it has not seen before. This provides an objective assessment of the generalizability of the model. Validation data is used to fine-tune the model, which in turn helps to select appropriate hyperparameters.

### Training algorithm

The training process is an important part of a feedforward neural network’s ability to learn from data and improve its predictive capabilities. This is achieved by optimizing the parameters of the network, which is achieved through training. In this section, the main focus is on using the BFGS quasi-Newton algorithm to train the loss function given by Eq. (8). The BFGS algorithm combines the advantages of the Newtonian technique, which shows fast convergence around the minimum, with the computational efficiency of the quasi-Newtonian approach. The goal of training the neural network is to minimize this mean squared error. It starts by initializing the weight vector $\vec {W}$ to a suitable starting point and the inverse Hessian approximation matrix $\vec {B}$ to an identity matrix or another positive definite matrix. The algorithm then computes the partial gradients of the loss function with respect to the weights and biases using backpropagation.

9 $$\begin{aligned} \frac{\partial J}{\partial w_{i j}^{(L)}} \, \text { and } \, \frac{\partial J}{\partial b_i^{(L)}}. \end{aligned}$$

The gradients show the direction as well as the magnitude of the weight and bias modifications needed to reduce the mean squared error. The BFGS Quasi-Newton algorithm modifies the weights and biases after computing the gradients in order to reduce the loss function. The inverse Hessian approximation matrix $\vec {B}$ is used with the gradients to determine the search direction. The proper step size for weight updates is determined via a line search. Finally, using the chosen step size and search direction, the weights and biases are updated. For a predetermined number of epochs or until a convergence condition is satisfied, the training process iterates through the phases of forward propagation, loss calculation, gradient computation, and weight updates. The mean squared error dropping below a certain threshold or exceeding a specified number of repetitions are two scenarios of the convergence criteria. The pseudo code for the working procedure of the designed FFBPN-BFGS Quasi-Newton algorithm is given in Table 1. All calculations and evaluations for this research were conducted on an HP laptop Elitebook 840 G2 with intel(R) Core(TM) i5-5300 CPU @ 2.30 GHz, 8.00 GB RAM, 64 bit operating in Microsoft Windows 10 Education edition running the R2018a version of MATLAB.

**Table 1:**
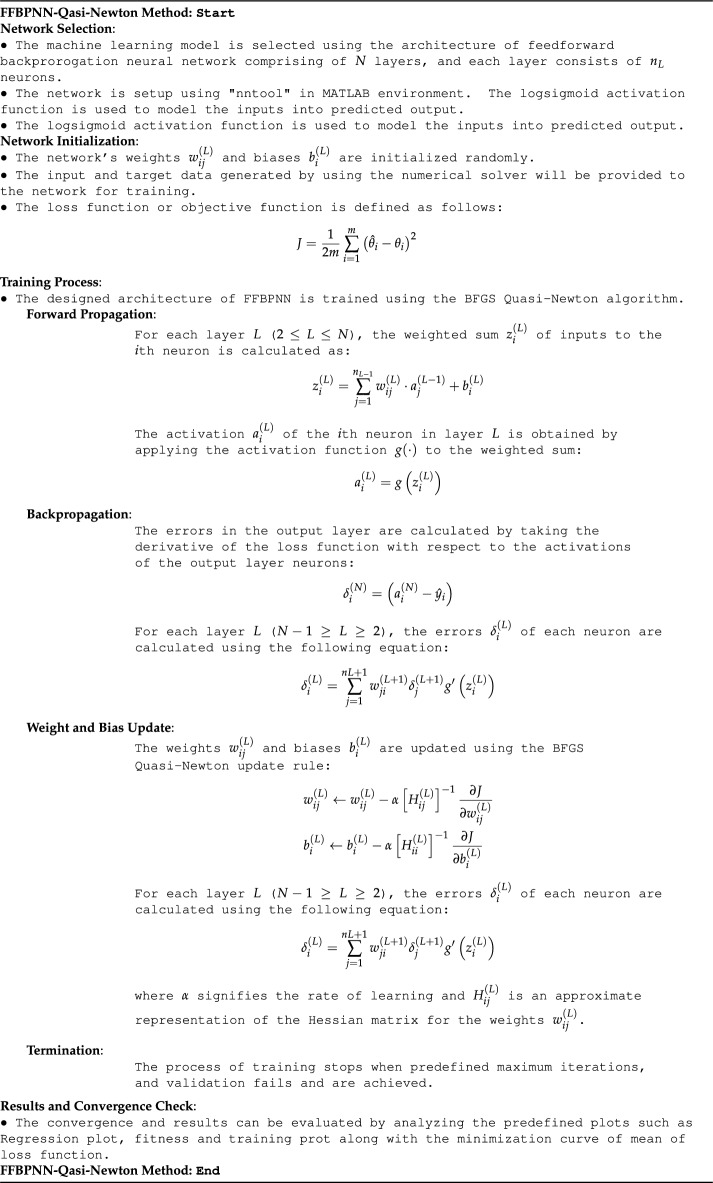
Pseudocode representing the operational procedures of a feedforward backpropagated neural network and the BFGS Quasi-Newton algorithm.

## Results and discussion

**Figure 3:**
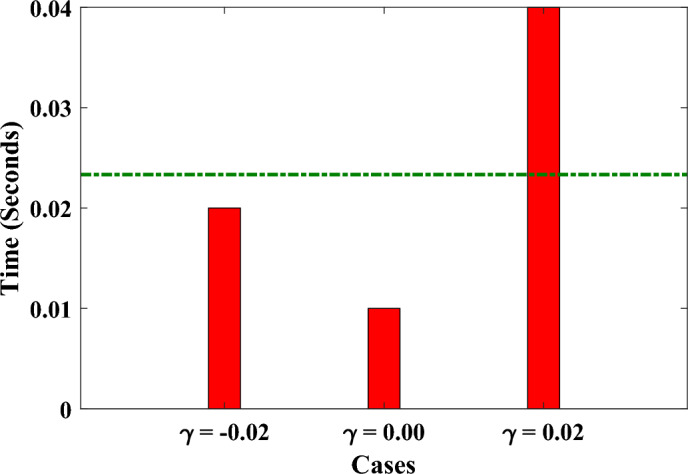
Time taken by the design technique for three different cases ($\gamma = -0.2, 0.0$ and 0.2) with fixed values of the other parameters such as $N_c = 0.5, m_1 = 1.0, N_r = 0.1225, E_G = 0.1, \theta _S = \theta _S= 0.5$. Green lines shows the mean time (0.0233 s).

**Figure 4:**
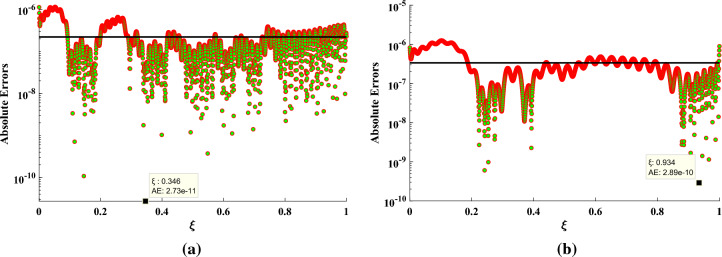
(**a**, **b**) Absolute error assessment: analytical solutions and FFBPNN-BFGS Quasi-Newton algorithm.

In this section, we have implemented the designed FFBPNN-BFGS Qasi Newton algorithm to study the rate of heat transfer in annular fin by varying different physical parameters. The solutions obtained by the designed algorithm are compared with some recently used techniques such as Finite difference method, Finite element method and homotopy perturbation method^29^. Table  2 presents values of the heat transfer rate at the tip of the annular fin achieved by different methods (FDM, FEM, HPM, RK-4, FFBPNN-BFGS QN) for three different cases ($\gamma = -0.2, 0.0$ and 0.2) with fixed values of the other parameters such as $N_c = 0.5, m_1 = 1.0, N_r = 0.1225, E_G = 0.1, \theta _S = \theta _S= 0.5$. In each case the FFBPNN-BFGS Qasi Newton algorithm achieves the value which is very close to the analytical solutions presented by the RK-4 method. The percentage error between the analytical solutions and designed method solutions for each case are $0.08\%$, $0.01\%$ and $0.004\%$ respectively. The time (seconds) taken by the algorithm to calculate solutions for these cases are shown in Fig. 3. Furthermore, the graph of absolute errors for two different cases $N_c = 0.5, m_1 = 1.0, \gamma = -0.2, N_r = 0.1225, G =0.025, E_G = 0.1, \theta _S = \theta _S= 0.4$ and $N_c = 0.4472, m_1 = 1.0, \gamma = -0.2, N_r = 0.0294, G = 0.016, E_G = 0.1, \theta _S = \theta _S= 0.5$ for 2001 data points $\in [0,1]$ are shown in Fig.  4. The minimum values for each case are $2.37\times 10^{-11}$ and $2.89\times 10^{-10}$ while the mean values for each case lies between $10^{-6}$ to $10^{-8}$ and $10^{-6}$ to $10^{-7}$ respectively. The FFBPNN-BFGS QN algorithm demonstrates an exceptional level of accuracy. This demonstrates the algorithm’s potential as a useful tool for analyzing heat transfer and other phenomena where precise numerical approximations are critical, such as when trying to predict the behavior of a system.

**Table 2:** Heat transfer values at the tip of the annular fin: a comparative analysis of FDM, FEM, HPM, and FFBPNN-BFGS-Quasi Newton method.

	FDM	FEM	HPM	RK-4	FFBPNN-BFGS QN
$\gamma =-0.2$	0.9090	0.9089	0.9115	0.90891186	0.908912602
$\gamma =0.0$	0.9134	0.9132	0.916	0.91315579	0.913156175
$\gamma =0.2$	0.9170	0.9168	0.9205	0.91675382	0.916754205

**Figure 5:**
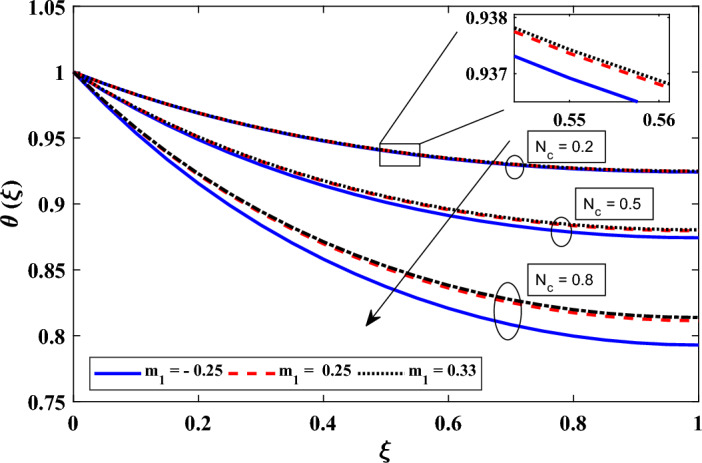
Investigating the influence of thermo-geometric parameter ($N_c$) and exponent of variable convective heat transfer coefficient ($m_1$) on dimensionless temperature distribution.

In addition, we investigate in detail the effect of various variables on the dimensionless heat transfer of annular fins. Specifically, we focused on understanding the consequences of a change in surface emissivity, heat transfer coefficient exponent, heat production, thermal conductivity variation, and coefficients between conduction and radiation processes. Figure 5 illustrates the influence of the thermo-geometric parameter ($N_c$) and the exponent of the convective heat transfer coefficient ($m_1$) on the temperature field. It was found that $N_c$ had an impact on local temperatures, with a lower value of $N_c$ corresponding to a faster rate of conductive heat transfer and, thus, higher local temperatures. This relationship was attributed to the inverse correlation between Nc and thermal conductivity. The material’s thermal conductivity is enhanced as $N_c$ drops, improving the conductive heat transfer inside the annular fin. Heat conduction through the fin increased as a result, leading to greater temperatures in the vicinity. Moving on to the variations in $m_1$, which represents different heat transfer modes with selected values of $m_1$ = -0.25, 0.25, and 0.33 representing laminar film condensation, laminar natural convection, and turbulent natural convection, respectively. It was observed that higher values of $m_1$ were associated with higher local temperatures across all $N_c$ values. This phenomenon can be understood by examining the physical mechanisms related to the convective heat transfer coefficient. In general, a higher value of $m_1$ indicated more intense convection, wherein the fluid flow exerted a greater impact on heat transfer. This intensified convection facilitated an increased transfer of heat from the fin surface to the surrounding fluid, leading to higher temperatures in the local region.

**Figure 6:**
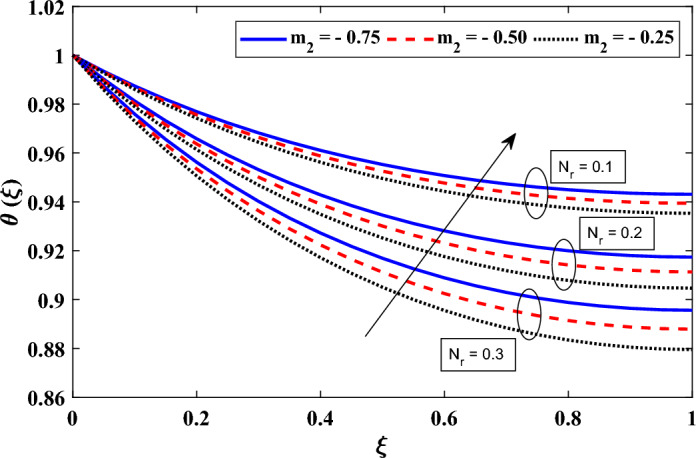
Exploring the impact of $N_r$ and exponent of variable emissivity ($m_2$) on dimensionless temperature distribution.

Figure 6 illustrates the impact of the conductive radiative parameter ($N_r$) and the exponent of the variable emissivity parameter ($m_2$) on the temperature field. The findings show that $N_r$ significantly affects the temperature distribution. The local temperature distribution diminishes as $N_r$ rises. With higher values of $N_r$, the surface loses more heat, which explains this phenomenon. The fin loses radiative heat more significantly when exponent of the variable radiative parameter rises, which lowers the nearby area’s temperature. Additionally, we noticed that when $m_2$ rises, the local temperature drops. This can be explained by the fact that surface emissivity increases together with $m_2$. The local temperature decreases as a result of faster energy emission caused by higher surface emissivity. As the radiated energy cannot be more in the low-temperature zone than at the base of the fin, the value of $m_2$ must be negative. The energy emitted from the fin surface behaves as expected because to this physical restriction.

**Figure 7:**
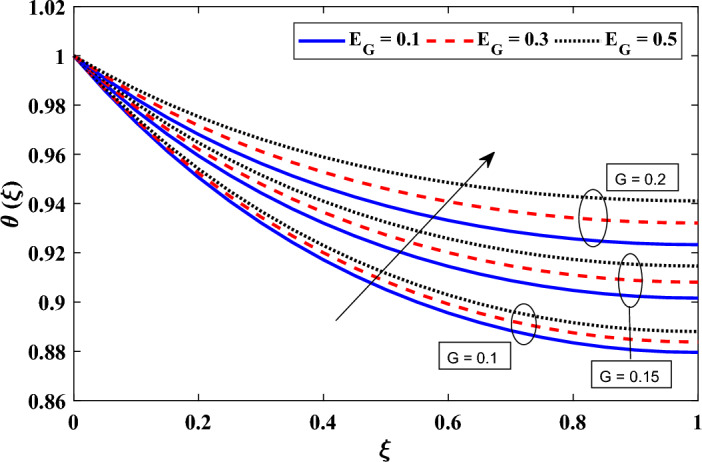
Exploring the relationship between two parameters: the heat generation parameter and the coefficient describing the variation of heat generation, and their influence on the distribution of dimensionless temperature.

In Fig. 7, we present the temperature distributions obtained for different values of the heat generation parameter (*G*) and the coefficient describing the variation of heat generation ($E_G$). In particular under steady-state conditions, higher values of the heat generation parameter (*G*) and the coefficient describing the variation of heat generation ($E_G$) cause an increase in the local temperature field within the annular fin due to the increased dissipation of heat to the surrounding environment. Indicated by an increase in the heat generation parameter (*G*), the annular fin is producing more heat internally. A greater internal temperature of the fin results from this enhanced heat output. Due to the increased temperature differential between the fin and its surrounds, heat transmission from the fin to the surroundings is accelerated. As more heat is transferred from the fin to the surroundings, the local temperature field rises as a result. Similar to how it shows that the rate of heat production within the fin is fluctuating more quickly as the coefficient representing the variance of heat generation ($E_G$) increases. The heat transmission process is made much more intense by this difference in heat output. Temperature changes are brought on by changes in heat production inside the fin, which occur more often. As a result of the system adjusting to the variations in heat generation, the local temperature field within the fin rises.

**Figure 8:**
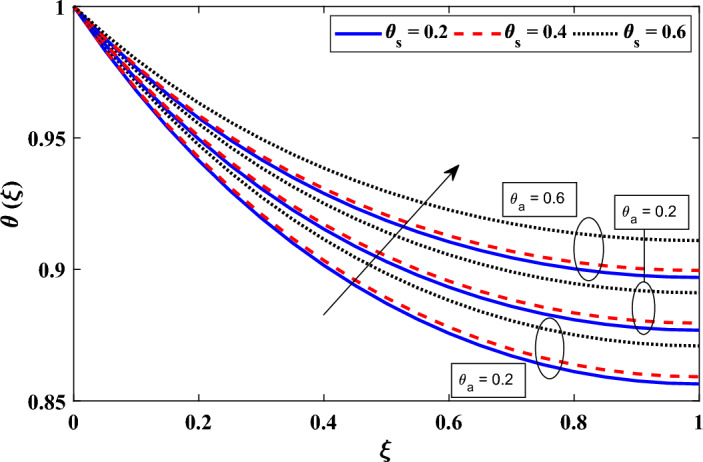
Examining the variations in $\theta _a$ and $\theta _s$ on the distribution of dimensionless temperature.

In order to control a system’s or device’s temperature and make sure it stays below the maximum permissible temperature, it is standard practice to use a heat sink. By facilitating the transmission of heat from the object to the surrounding area, heat sinks are intended to improve heat dissipation and avoid overheating. Figure 8 shows the plots of the nondimensional temperature field for various values of the convective sink temperature ($\theta _a$) and the radiative sink temperature ($\theta _s$) along the radial direction. An increase in a and s in either scenario causes the surrounding temperature to rise. The impact of $\theta _a$ and $\theta _s$ on the heat transfer processes, notably convective and radiative heat transfer, is the cause of this temperature increase. The difference in temperature between the device and the sink decreases as $\theta _a$ or $\theta _s$ rises. As a result, there is a reduction in the rate of heat loss via convective and radiative processes. A greater local temperature results from more heat being held inside the device as a result of the decrease in heat loss. The research also shows that the radiative sink temperature ($\theta _s$) has a far less impact than the convective sink temperature ($\theta _a$). This finding implies that the convective heat transfer mechanism has a higher influence on the device’s ability to regulate temperature than the radiative heat transfer method. To put it another way, modifications to a have a more significant impact on the local temperature field.

### Performance analysis

**Figure 9:**
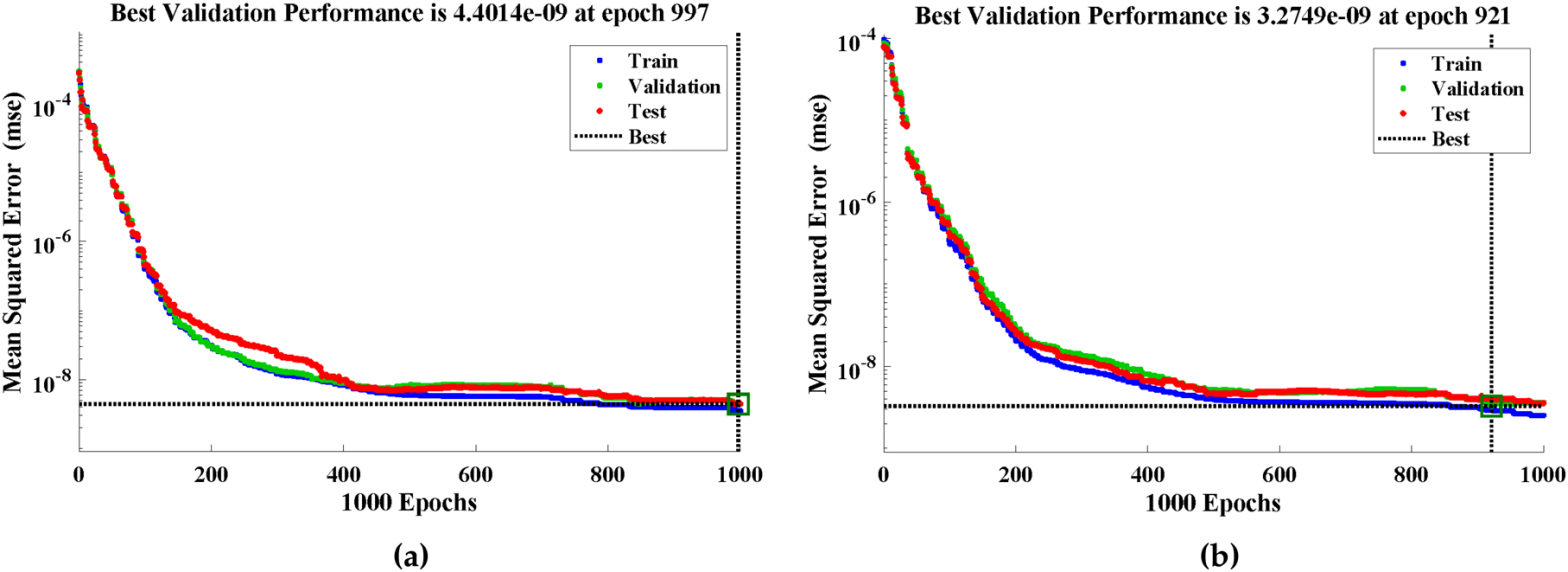
(**a**, **b**) Assessments of the convergence of performance value in terms of mean square error.

In this section, we present a comprehensive performance analysis of the designed algorithm, showcasing its effectiveness in calculating the solutions for the heat transfer problem of annular fin. The performance plots of the designed technique for different cases generated by the nntool provide valuable insights into the algorithm’s capabilities and its ability to approximate the desired outcomes. The performance plots shown in Fig.  9 offer a visual representation of mean square error between the targeted and predicted values to assess the algorithm’s performance during the training process. With a best validation performance of $4.4014\times 10^{-09}$ and $3.2749\times 10^{-09}$ at epoch 997 and 921 respectively, the algorithm performs exceptionally well. This suggests that the algorithm’s predictions closely match the desired values, leading to a little difference between the expected and actual results. Such an impressive result demonstrates the algorithm’s excellent accuracy and dependability in determining solutions to the annular fin heat transfer problem.

**Figure 10:**
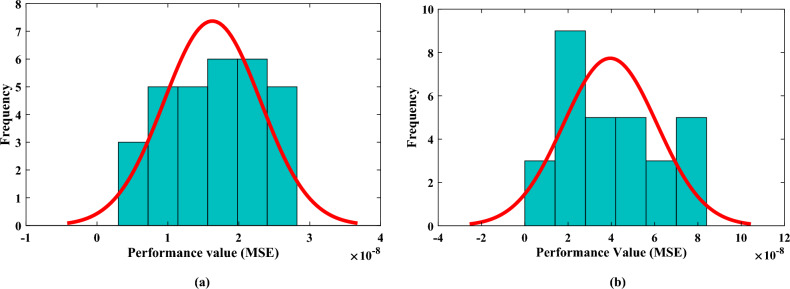
(**a**, **b**) Histogram fits with normal distribution curves for the performance value achieved during the multipe executions of the designed technique.

Furthermore, the designed FFBPNN-BFGS Qasi Newton method is executed for 30 independent runs to extend the a comprehensive performance analysis. The best performance values obtained from these runs were collected and analyzed. To provide insights into the distribution and characteristics of these performance values, histograms with corresponding fits were constructed. The histogram represents the frequency distribution of the values, allowing us to analyze the distribution pattern and identify any underlying trends or patterns as shown in Fig.  10. It is interesting to note that the majority of the performance values appear to be concentrated in the middle range of the x-axis, with fewer occurrences at the extremes. This suggests that the algorithm’s performance is generally consistent and achieves relatively good results, as indicated by the concentration of values around the middle range. In each case, the histogram fit revealed a distribution of performance values. The minimum performance values observed was $5.03641\times 10^{-09}$, $8.73119\times 10^{-09}$ while the mean and median values were $1.92607\times 10^{-08}$, $8.73119\times 10^{-09}$ and $1.75305\times {-08}$, $3.40955\times 10^{-08}$, respectively. This indicates that the algorithm exhibited good performance with variations across the runs.

**Figure 11:**
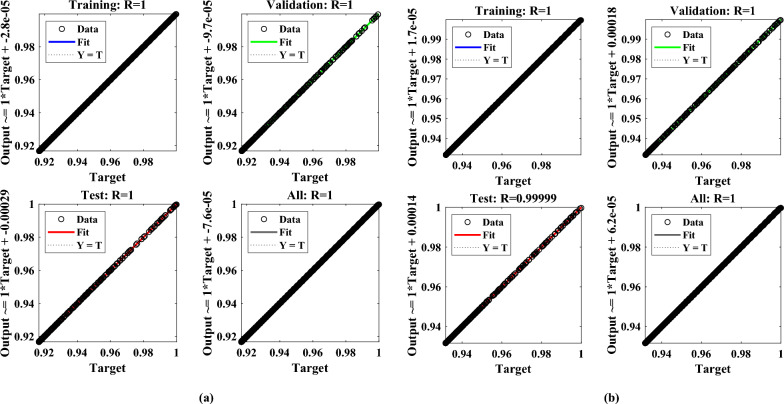
(**a**, **b**) Exploring regression performance: comparing predicted and actual values.

Finally, the regression plots are shown through Fig.  11 to compare the targeted (expected values) and the predicted values generated by the designed technique. It can be observed that values of regression ($R^2$) are exactly 1 that shows the well-fitted regression highlighting a tight clustering of points along a diagonal line indicating the strong correlation between the target and predicted values.

## Conclusion

In this work, the efficiency of an annular fin made of a functionally graded material (FGM) for increasing heat transmission in diverse applications was investigated. We have looked into the heat transport properties and temperature distribution within the annular fin using a supervised machine learning approach, more especially the feedforward backpropagated architecture with the BFGS Quasi-Newton training algorithm. We have verified the precision and dependability of our findings by comparing them to those obtained using more established techniques, including the homotopy perturbation method, the finite difference approach, and the Runge-Kutta method. Our investigation has revealed useful information about how the annular fin performs under various physical conditions, confirming its efficiency in improving heat transmission. Some important outcomes are analyzed as:

- The thermo-geometric parameter ($N_c$) has a significant impact on local temperatures in annular fins, with lower $N_c$ values resulting in faster conductive heat transfer and higher local temperatures due to the inverse correlation between $N_c$ and thermal conductivity.
- Higher values of $m_1$ indicate more intense convection, leading to increased heat transfer from the fin surface to the surrounding fluid and higher temperatures in the local region.
- The local temperature drops as $m_2$ rises, indicating that the energy emitted from the fin surface aligns with physical restrictions, where the radiated energy cannot be higher in the low-temperature zone compared to the base of the fin.
- The conductive radiative parameter ($N_r$) has a significant impact on the temperature distribution in annular fins, with higher $N_r$ values leading to a decrease in local temperature due to increased heat loss from the surface.
- Higher values of the heat generation parameter (*G*) and the coefficient describing the variation of heat generation ($E_G$) lead to an increase in the local temperature field within the annular fin due to enhanced dissipation of heat to the surrounding environment.
- The local temperature field within the fin rises as the system adjusts to variations in heat generation caused by changes in the heat production process inside the fin.

Looking ahead, our future research endeavors will focus on leveraging advanced machine learning techniques to design FGMs with tailored grading profiles aimed at maximizing heat transfer efficiency. By delving deeper into the optimization of FGM structures, we aim to push the boundaries of thermal engineering and contribute to the development of innovative solutions for diverse applications requiring enhanced heat transmission capabilities.

## Data Availability

The datasets used and/or analysed during the current study available from the corresponding author on reasonable request.
